# Cost-Analysis Of A Geriatrician-Led Falls Prevention Clinic Among Older Adults At High Risk Of Future Falls

**DOI:** 10.1093/geroni/igaf122.3461

**Published:** 2025-12-31

**Authors:** Jennifer C Davis, Elise Wiley, Larry Dian, Kenneth Madden, Karim Khan, Naaz Parmar, Katrina Loutet, Teresa Liu-Ambrose

**Affiliations:** The University of British Columbia, Vancouver, British Columbia, Canada; The University of British Columbia, Vancouver, British Columbia, Canada; The University of British Columbia, Vancouver, British Columbia, Canada; The University of British Columbia, Vancouver, British Columbia, Canada; The University of British Columbia, Vancouver, British Columbia, Canada; The University of British Columbia, Vancouver, British Columbia, Canada; The University of British Columbia, Vancouver, British Columbia, Canada; The University of British Columbia, Vancouver, British Columbia, Canada

## Abstract

A geriatrician-led Falls Prevention Clinic is a best practice and evidence-based approach for falls prevention that has demonstrated feasibility and acceptability. The economic impacts of this approach, which directly impact its sustainability within the healthcare system, remain unknown. Our primary objective was to determine the costs and potential savings associated with a multi-disciplinary geriatrician-led Falls Prevention Clinic among older adults at high risk of falls. Operating costs of the Falls Prevention Clinic were determined based on operating costs (i.e., personnel, equipment, overhead), medical services plan costs, and personnel costs. Falls Prevention Clinic usage over 12 months was detailed based on monthly frequencies of new and repeat visits. Longitudinal health resource utilization was ascertained over 12 months. Cost savings from falls averted over 12 months were estimated. Main outcome measures included: operating costs of the clinic, estimated annual health resource utilization savings, number of falls over 12 months and health care costs (i.e., health care practitioner, hospital admissions, laboratory tests/investigations). A total of 543 patients were seen over a year, with 240 new and 303 follow-up patients. The total direct health resource utilization costs were 4,892 (7,767) (2024 CAD$) per person over 12 months. The annual estimated cost-saving of the clinic from fall prevention is 956,288 (2024 CAD$). The Fall Prevention Clinic provides a multi-disciplinary approach that is best practice, evidence-based for fall prevention. This approach has demonstrated feasibility and effectiveness and saves health care dollars; thus, it is an effective and economically attractive approach to consider for implementation.

